# A deep learning approach for acoustic-based identification of muscle tension dysphonia and spasmodic dysphonia

**DOI:** 10.3389/fdgth.2026.1690534

**Published:** 2026-07-09

**Authors:** Zhou Zhou, Yuan Cheng, Qingyi Ren, Yike Li, Xu Yuanyue, Jing Kang, Cheng Lu, Pingjiang Ge

**Affiliations:** 1 Department of Otolaryngology Head Neck Surgery, Guangdong Provincial People's Hospital, Guangdong Academy of Medical Sciences, Southern Medical University, Guangzhou, China; 2 Department of Otolaryngology Head Neck Surgery, Shenzhen People's Hospital (The Second Clinical Medical College, Jinan University, The First Affiliated Hospital of Southern University of Science and Technology), Shenzhen, China; 3 Department of Radiology, Guangdong Provincial People's Hospital, Guangdong Academy of Medical Sciences, Southern Medical University, Guangzhou, China; 4 Medical Research Institute, Guangdong Provincial People's Hospital, Guangdong Academy of Medical Sciences, Southern Medical University, Guangzhou, China; 5 Guangdong Provincial Key Laboratory of Artificial Intelligence in Medical Image Analysis and Application, Guangzhou, China; 6 Department of Otolaryngology-Head and Neck Surgery, Vanderbilt University Medical Center, Nashville, TN, United States

**Keywords:** acoustic, deep learning, diagnosis, muscle tension dysphonia, spasmodic dysphonia

## Abstract

**Problem:**

Differentiating between spasmodic dysphonia (SD), a neurological disorder, and muscle tension dysphonia (MTD), a behavioral voice disorder, based on auditory perception alone is a common clinical challenge. This diagnostic difficulty can lead to delays in appropriate treatment, highlighting the need for objective and reliable assistive diagnostic tools.

**Aim:**

This study aims to develop and validate an artificial intelligence (AI) model based on deep learning to automatically differentiate between healthy voices, SD, and MTD using only voice audio recordings, and to compare its diagnostic performance against human experts.

**Methods:**

A retrospective analysis was conducted on 1,597 voice samples (595 healthy, 471 MTD, 531 SD). Voice audio was processed into Log-Mel spectrograms. Pre-trained convolutional neural networks (CNNs), including VGG16, ResNet50, and DenseNet161, were employed for transfer learning to perform both binary (Healthy vs. Disordered) and ternary (Healthy vs. MTD vs. SD) classification. The model's performance was evaluated on a held-out test set and compared to the diagnostic assessments of four otolaryngologists.

**Results:**

The AI model achieved an accuracy of 89.5% (AUC = 0.956) in distinguishing healthy voices from disordered ones. For the more complex ternary classification, the model attained an accuracy of 71.6%, with class-specific AUCs of 0.957 (Healthy), 0.731 (MTD), and 0.855 (SD). This performance surpassed that of human experts, who achieved average accuracies of 78.2% in binary classification and 60.6% in ternary classification on the same test set.

**Conclusion:**

The deep learning model trained on a Mandarin pathological voice dataset achieves favorable performance in distinguishing SD from MTD using only voice audio signals, with classification results comparable to those of experienced clinical specialists. This technology serves as a promising objective auxiliary tool for the preliminary screening and differential diagnosis of voice disorders.

## 1 Introduction

Spasmodic dysphonia (SD) and muscle tension dysphonia (MTD) represent two clinically significant voice disorders that pose considerable diagnostic challenges. SD is a focal laryngeal dystonia characterized by involuntary vocal fold spasms, leading to strained voice quality and intermittent voice breaks (1, 2). In contrast, MTD is a functional disorder resulting from excessive laryngeal and paralaryngeal muscle tension, often associated with psychological factors, vocal misuse, or compensatory mechanisms (3–5). Despite their distinct pathophysiologies, these conditions frequently exhibit overlapping perceptual features and nearly indistinguishable laryngoscopic findings, particularly in cases with atypical presentations (6–11). This diagnostic ambiguity is concerning given their fundamentally different treatment pathways: while SD typically requires botulinum toxin injections to alleviate involuntary spasms, MTD responds well to voice therapy alone (12–16). Misdiagnosis can therefore lead to delayed appropriate treatment, unnecessary procedures, and potential harm to patients.

Current artificial intelligence (AI) technologies have demonstrated remarkable capabilities in healthcare diagnostics, particularly in image-based recognition systems (17–20). However, the visual similarity between SD and MTD in laryngeal imaging limits the utility of computer vision approaches for their differentiation. Instead, acoustic analysis offers a more promising avenue, as these disorders exhibit distinguishable vocal characteristics despite their perceptual similarities. This study leverages this acoustic distinction to develop an AI-based diagnostic tool using voice samples.

While numerous machine learning studies have addressed pathological voice detection, most existing frameworks are limited to binary classification—distinguishing normal from pathological voices or differentiating among various voice disorders vs. normal controls (21–25). To our knowledge, no previous research has implemented a three-class classification system specifically designed to discriminate between SD, MTD, and normal voices. Our work fills this critical gap by developing a tripartite deep learning model that not only identifies pathological voices but also enables differential diagnosis between these two commonly confused disorders.

To address these challenges, we propose a convolutional neural network (CNN)-based framework trained on retrospectively collected voice samples from confirmed SD and MTD patients, along with normal controls. Our system introduces several key innovations:

- Three-Class Classification Architecture: A novel deep learning framework specifically designed for simultaneous differentiation of SD, MTD, and normal voices, addressing a critical gap in current literature.
- Dual Diagnostic Capability: The model serves both screening (normal vs. pathological) and differential diagnostic (SD vs. MTD) functions within a unified system.
- Clinical Workflow Integration: The design prioritizes practical implementation in otolaryngology and speech-language pathology settings, with rapid processing times compatible with clinical workflows.
- Interpretable Feature Analysis: Implementation of explainable AI techniques to identify salient acoustic features driving classification decisions, enhancing clinical trust and adoption.
- Robust Performance Validation: Comprehensive evaluation using multiple metrics (accuracy, F1-score, recall, precision, ROC-AUC) against established machine learning benchmarks.
- Foundation for Future Development: The architecture supports integration with emerging technologies and additional voice disorders, providing an extensible platform for voice diagnostics.

This paper is structured as follows: Section 2 reviews related work in the use of deep learning in the detection of voice disorders; Section 3 details our methodology and dataset characteristics; Section 4 presents experimental results and model performance; Section 5 elaborates on the clinical implications of this study, along with its limitations and prospects for future research;and Section 6 summarizes the overall findings of this work.

## 2 Related works

Artificial intelligence (AI) is revolutionizing the field of medicine by providing powerful tools for patient data analysis (26). As a subset of AI, deep learning utilizes multi-layered neural networks to automatically learn from data and can perform complex tasks such as image recognition, speech recognition, and natural language processing (27). These neural networks have evolved from earlier forms of machine learning and gained rapid popularity in recent years. Compared with other machine learning algorithms, multi-layered neural networks exhibit superior performance in analyzing and learning from unstructured data, reduce the need for human supervision, and possess the flexibility to adapt to a variety of use cases (28).

In the field of laryngology, deep learning models achieve the classification of normal and pathological voices by analyzing acoustic signals, and further categorize various types of voice disorders, including vocal nodules, polyps, unilateral vocal fold paralysis, and vocal fold sulcus (21). Beyond diagnostic classification, an AI-driven clinical decision support system (CDSS) has been proposed, which can significantly improve the accuracy of patient diagnosis (29). With technological advancements and the increasing availability of large-scale datasets, the application of deep learning in laryngology has become increasingly important, holding the potential to completely revolutionize the diagnosis and treatment of voice disorders.

Related research in past 5 years is listed in Table 1. Chen and Chen (30) proposed an automatic diagnostic method for pathological voices based on deep neural networks (DNNs). This method can learn high-level features from raw acoustic characteristics and distinguish pathological voices from healthy ones, achieving an accuracy of 98.6%. Chen et al. (31) retrospectively collected recordings of sustained phonations of/a/ and/i/ from a clinical database and verified that the model using Mel-spectrograms exhibited the best performance, with a binary classification accuracy of 92%. Hung et al. (32) developed an interpretable SincNet system for classifying or detecting pathological voices, which was tested on three different speech datasets from Far Eastern Memorial Hospital. The test results demonstrated that the proposed SincNet system could effectively provide supervisor-recognized accuracy and sensitivity when predicting input pathological waveforms. Zakariah et al. (33) designed a DNN that was trained on the SVD database to generate a highly accurate speech-based disease prediction model. This model used audio files of vowels/a/,/i/,/u/, and “continuous sentences” to evaluate the generalization ability of the developed model to completely new data, achieving a maximum binary classification accuracy of 77.49%. Both Wang et al. (34) and Zhang et al. (35) constructed deep learning models through autoencoder training. Trained on the vowel/a/ from different databases, this method not only achieved high intra-corpus and cross-corpus classification accuracy but also generated embeddings sensitive to speech quality with good robustness across different corpora. Mahmood (36) developed a comprehensive model that employs various deep learning techniques to improve the detection of voice pathologies. In this regard, as a means of extracting these features, a state-of-the-art approach combining Gammatonegram features with Scalogram Teager-Kaiser Energy Operator (TKEO) features is proposed, and the proposed feature extraction scheme is named Combine Gammatonegram with (TKEO) Scalogram (CGT Scalogram). For binary classification, the model achieved an accuracy of 96%, while the accuracy for multi-class classification reached 94.4%. Jegan and Jayagowri (37) proposed an optimized convolutional neural network (CNN)-based framework for speech disorder detection, with its core novelty lying in the development of a CNN model optimized via the Artificial Bee Colony (ABC) algorithm. Experiments on three databases containing normal and pathological voice signals showed that this framework outperformed conventional CNN networks in sensitivity, precision, specificity, F1-score, and accuracy, achieving promising detection performance. Additionally, the explainable artificial intelligence (XAI) algorithm based on Gradient-weighted Class Activation Mapping (GRAD-CAM) further validated the effectiveness of the decision-making process of the proposed optimized CNN model. Meanwhile, Ma et al. (38) developed machine learning models—eXtreme Gradient Boosting (XGBoost) and Light Predict-Boosting Machine (LightGBM)—trained for classifying benign and malignant lesions and predicting malignant tumors. Using clinically collected acoustic data, the XGBoost model exhibited high diagnostic value in distinguishing between benign and malignant vocal fold lesions, with age and specific acoustic parameters identified as key predictive factors in the model.

**Table 1:** Summary of related works on AI models based on acoustic for voice disorders diagnosis (past 5 years).

Reference	Years	Database	Learning method	Samples	Classification	Accuracy
Chen and Chen2 (30)	2020	VOICED	Autoencoder	Vowel/a/	2(healthy vs. pathological)	0.986
Chen et al. (31)	2022	Personal	CNN	Vowels:/a/ and/i/	2(healthy vs. pathological)	0.92
Hung et al. (32)	2022	FEMH	CNN	Vowel/a/	4(neoplasm, functional dysphonia, vocal palsy, and phonotrauma)	0.77
Zakariah et al. (33)	2022	SVD	MLP	Vowels and sentence	2(healthy vs. pathological)	0.77
Wang et al. (34)	2023	VOICED	Autoencoder	Vowel/a/	2(healthy vs. pathological)	0.99
Zhang et al. (35)	2024	SVD	Autoencoder	Vowel/a/	2(healthy vs. pathological)	0.826
Mahmood (36)	2024	SVD	CNN	Vowel/a/	2(healthy vs. pathological) 5 pathological voices	0.96 0.944
Jegan and Jayagowri (37)	2024	SVD, AVPD and VOICED	CNN	Vowels	2(healthy vs. pathological)	0.978
Ma et al. (38)	2025	Personal	MLP	Vowels	2(benign vs. malignant vocal cord lesions)	0.92

In summary, previous work has laid a solid foundation for the application of AI tools in voice disorder prediction. However, existing studies have obvious limitations. Most prior studies utilized general-purpose databases, with few employing clinically collected data. The classification labels in most studies were limited to the binary distinction between normal and pathological voices. Moreover, these studies, predominantly from the engineering field, focused on algorithm improvement to enhance model stability and accuracy, rather than addressing practical clinical challenges.

To address these limitations, our study offers supplementary contributions with the following key features. First, all data used in our research are clinically collected and sourced from multiple hospitals, ensuring a certain level of data generalization. Second, our research aims to serve clinical practice: we expect to develop an open-access diagnostic tool for the accurate diagnosis of Spasmodic Dysphonia (SD) and Muscle Tension Dysphonia (MTD), thereby facilitating subsequent clinical diagnosis and treatment.

## 3 Materials and methods

This study adheres to the principles of the Declaration of Helsinki and has been approved by the Ethics and Research Committees of Guangdong Provincial People's Hospital and Shenzhen People's Hospital. From December 2012 to April 2024, a total of 595 healthy voice audio samples and 1,002 pre-treatment voice disorder audio samples were collected from the Otolaryngology Departments of Guangdong Provincial People's Hospital and Shenzhen People's Hospital. The pre-treatment samples included MTD (*n* = 471) and SD (*n* = 531). All samples are clinically clearly diagnosed disease specimens, and those with other comorbidities are excluded. All patients and healthy individuals are aged between 18 and 80 years old with no gender restrictions. The samples were recorded at comfortable volume levels using Praat version 4.5, a Shure 8 microphone, in mono sound with a sampling rate of 44,100 Hz and 16-bit resolution. The data was saved in uncompressed.wav format. First, 1,597 samples were randomly divided into three groups in a 3:1:1 ratio, with 960 samples used as the training set, 313 samples as the internal validation set, and 324 samples as the test set. Through random grouping, 358 healthy voice audio samples, 282 MTD samples, and 320 SD samples were selected for the training set (see Table 2). We strictly adopted patient-level data splitting. All data of one patient were entirely assigned to either the training set, validation set or test set, with no patient crossover between different datasets.

**Table 2:** Grouping and labeling of vowel-a audio samples.

Class Index	Vowels-a		
	Training cohort	Validation cohort	Test cohort
Healthy	358	117	120
MTD	282	91	98
SD	320	105	106

Healthy, normal voice; MTD, muscle tension dysphonia; SD, spasmodic dysphonia.

### 3.1 Data preprocessing and model building

Convolutional Neural Networks (CNNs) in computer vision excel at handling two-dimensional image data. Therefore, we convert voice audio data into Log-Mel spectrograms by resampling to 44.1 kHz, applying pre-emphasis, framing, and windowing (window length = 1,024 samples, hop length = 512 samples), performing a 1,024-point FFT, and computing 64 Mel filter banks covering 0–22 kHz followed by logarithmic power conversion. These parameter settings follow standard practices in voice analysis and were verified through preliminary testing to yield stable model performance. No additional noise reduction was applied, as all recordings were obtained under controlled conditions. To address the issue of limited training dataset size, we employ SpecAugment technology, applying frequency and time masking to the original audio in the training set based on a predefined maximum mask ratio, thereby simulating voice audio data augmentation effects.

We employed different CNN architectures, such as VGG16, ResNet50, and DenseNet161 models, which were pre-trained on the ImageNet dataset for transfer learning. We categorized the pathological conditions into binary (Healthy vs. MTD&SD) and ternary (Healthy vs. MTD vs. SD) classifications, trained the CNNs, and used visualization tools like Grad-CAM to interpret the predictive models. For the final prediction of input instances, we utilized ensemble learning methods by calculating the average probability to obtain the labels.

**Figure 1:**
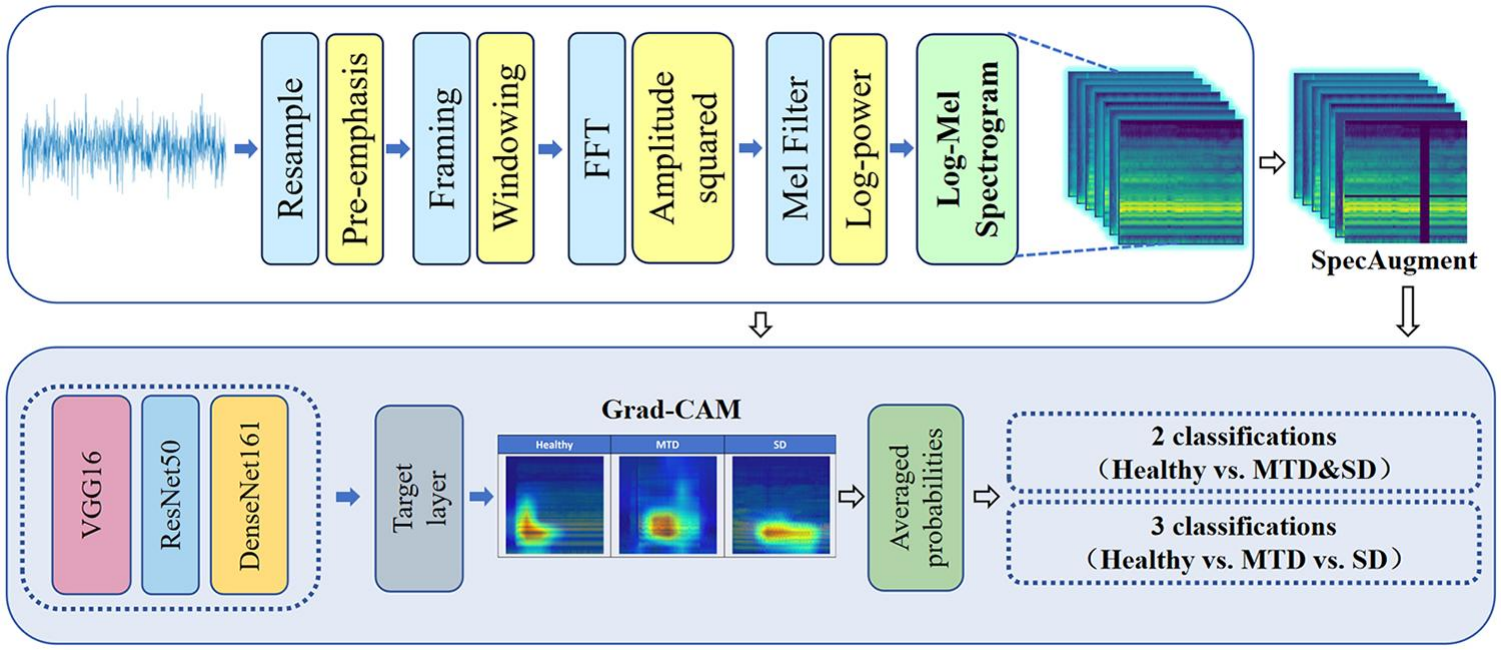
An overview of the AI framework. Healthy = normal voice; MTD, muscle tension dysphonia; SD, spasmodic dysphonia.

Regarding hyperparameter settings for model training, the training cohort was used for model optimization and the internal validation cohort was used for hyperparameter tuning and early stopping. For the ensemble framework, three CNN-based submodels (VGG16, ResNet50, and DenseNet161) were fine-tuned under identical configurations implemented in PyTorch. Each submodel was trained with a batch size of 4 for up to 200 epochs using the AdamW optimizer and a OneCycleLR scheduler. The scheduler parameters were set as follows: maximum learning rate = 9 × 10^‒7^, pct_start = 0.3, div_factor = 2, and anneal_strategy = “linear”. The cross-entropy loss function was used for optimization. To mitigate overfitting, an early stopping mechanism based on validation accuracy was applied, whereby training was automatically terminated when validation accuracy failed to improve for a predefined number of epochs. During inference, the softmax probabilities from the three submodels were averaged to generate the final ensemble prediction. The machine learning process was conducted using Python 3.9.1 and PyTorch 2.0.1 on Ubuntu 22.04. The technical roadmap is illustrated in the following Figure 1.

### 3.2 Generation of heat maps

**Figure 2:**
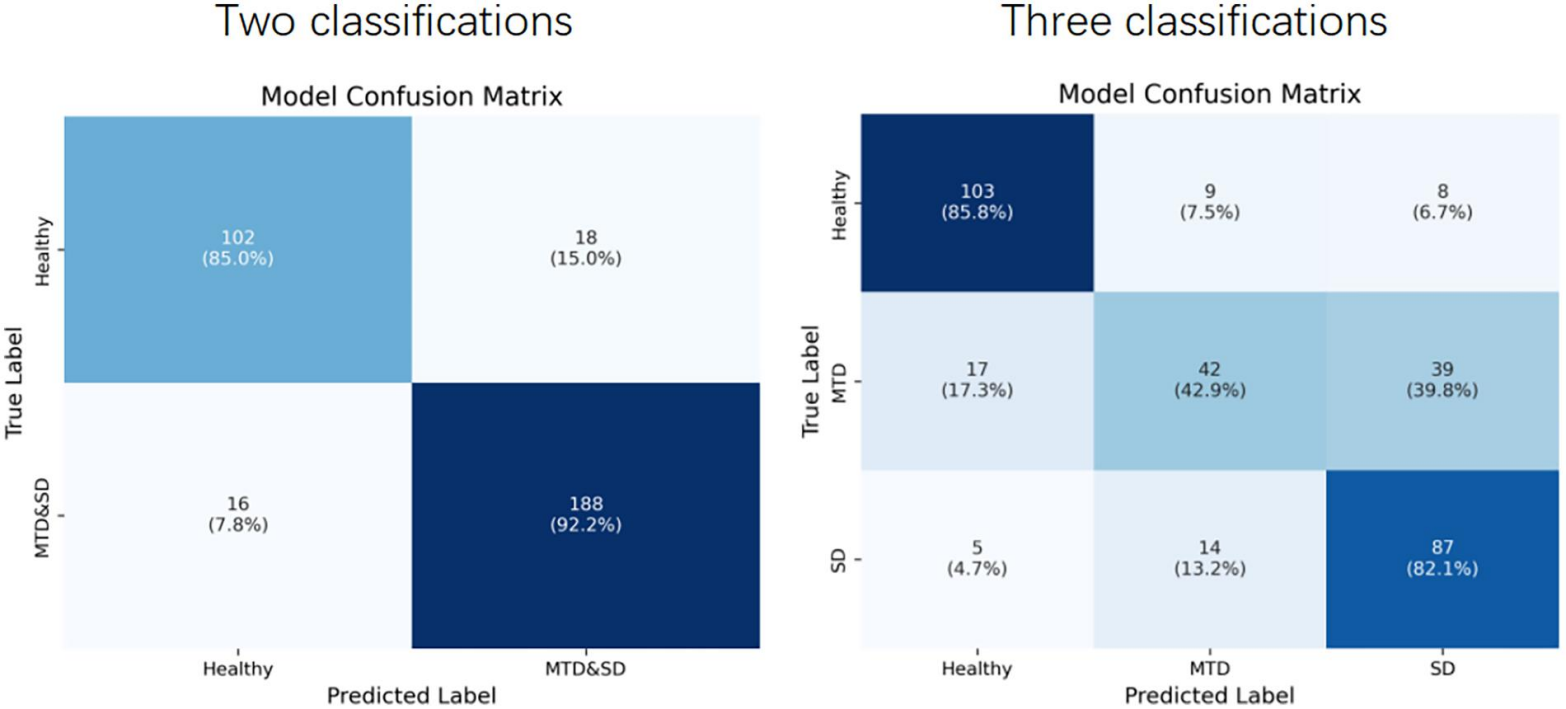
Confusion matrix of 2 and 3 classifications. Healthy = normal voice; MTD, muscle tension dysphonia; SD, spasmodic dysphonia.

Gradient-Weighted Class Activation Mapping was used tovisualize model's rationale for decision-making (Figure 2C). In essence, this approach leverages the gradients of the target class flowing into the final convolutional layer to produce a coarse localization heat map, highlighting the critical regions in the image (39). In this study, heat maps were generated in a 3D fashion and rescaled to match the original images using TensorFlow 2.11 in Python 3.91 (Python Core Team) (40).

### 3.3 Comparison with human experts

In clinical practice, the diagnosis of Spasmodic Dysphonia (SD) primarily relies on clinical professionals' auditory perceptual assessment—an inherently subjective process prone to variability due to differences in expertise and experience (41). To systematically evaluate the proposed AI model's accuracy and stability, a comparative analysis was conducted between the model and human experts using the same voice audio data.

Four specialists with diverse clinical backgrounds were enrolled to ensure representative clinical decision-making perspectives: two senior laryngologists (20 and 15 years of voice disorder diagnosis experience, respectively), one attending laryngologist (8 years of otolaryngology-head and neck surgery experience, focusing on voice pathology), and one speech-language pathologist (8 years of auditory perceptual evaluation experience for voice disorders).

The expert assessment used the same 324-sample test dataset as the AI model, including healthy controls (*n* = 120), Muscle Tension Dysphonia (MTD, *n* = 98), and SD (*n* = 106). All samples were anonymized (unseen by experts to avoid familiarity bias), and 15% of the test samples (*n* = 50, randomly selected and replicated) were incorporated to assess intrarater reliability (consistency of an expert's diagnoses over time). All samples were shuffled to prevent sequence bias and stored on a password-protected computer.

Each expert received only the audio samples (no additional clinical information, e.g., demographics or medical history) and was instructed to classify each sample into “Healthy”, “MTD”, or “SD”. A standardized electronic spreadsheet was provided for recording diagnoses, with clear instructions to minimize ambiguity. All procedures were approved by the institutional review board (IRB) to comply with research ethics, with sample anonymization and storage security verified.

### 3.4 Statistical analysis

Descriptive statistics were applied as appropriate. The overall predictability of a model was evaluated by the area under the receiver operating characteristic (AUROC) curve. The optimal cutoff threshold on the curve was determined at the point with minimal distance to the upper left corner on the training cohort and subsequently applied to the test cohort. The numbers of correctly and incorrectly classified cases were displayed in a confusion matrix, and these were used to calculate the performance metrics, including accuracy, recall, specificity, precision, and F1-score. These metrics offer comprehensive insights into the model's performance, covering overall correctness in identifying both positives and negatives(accuracy), sensitivity in detecting positive cases (recall), capability in ruling in patients (specificity), propensity for preventing false alarms (precision), and effectiveness in identifying positive cases while minimizing false positives and false negatives (F1-score). They were derived as shown in Textbox 1. Intrarater consistency was evaluated using the intraclass correlation coefficient (ICC). Differences in performance between models and between the models and human experts were assessed using the McNemar's test. The significance level was set at 0.05. Statistical analyses were conducted using Python 3.91 (40).

Textbox 1 The calculation of performance metrics.

**Table:** 

Accuracy = (True positive + True negative)/Total sample size
Sensitivity = True positive/(True positive + False negative)
Specificity = True negative/(True negative + False positive)
Precision = True positive/(True positive + False positive)
F1-score = 2 × True positive/(2 × True positive + False positive + False negative)

## 4 Results

**Figure 3:**
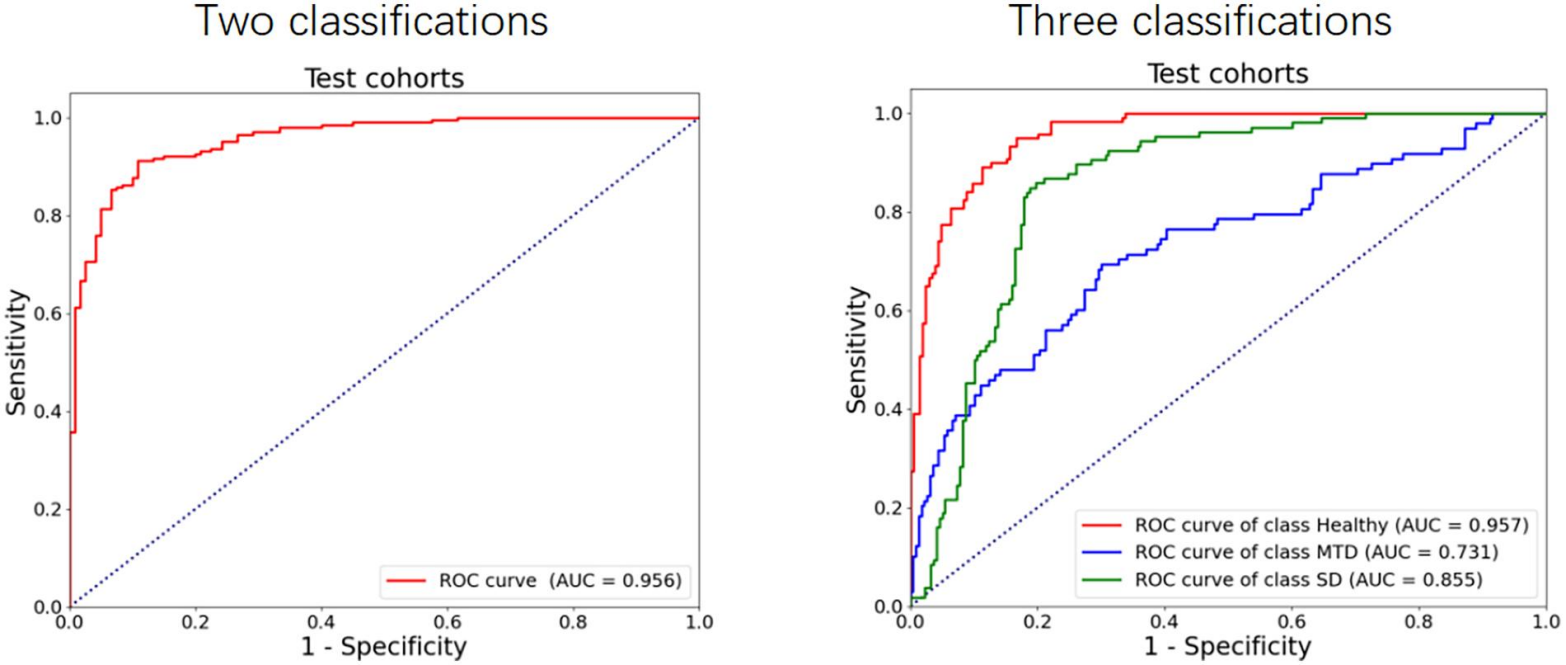
Receiver operating characteristic curves of 2 and 3 classifications. Healthy = normal voice; MTD, muscle tension dysphonia; SD, spasmodic dysphonia.

Table 3 presents the training results for the different classification methods, including 2 (Healthy vs. MTD&SD), 3 (Healthy vs. MTD vs. SD) different conditions trained by the CNN. The 2-classification condition could equally distinguish pathological voices from normal voices. In our model, the accuracy of pathological voice detection reached 89.5%; the sensitivity was 92.2%, specificity was 85%, and AUC was 0.956. Using the 3-classification condition, we aimed to differentiate spasmodic dysphonia and muscle tension dysphonia. The accuracy was 71.6%, sensitivity was 70.2%, specificity was 85.8%, and AUC was 0.855. The confusion matrix in Figure 2 intuitively presents the actual category distribution and misclassification patterns among different voice disorder groups, clearly showing how many samples are correctly classified and the detailed misclassification tendency between the two disease categories. Figure 3 shows the ROC curves of these results. The enrolled participants in this study were aged 18 to 80 years. To eliminate potential confounding effects attributed to age, we performed a subgroup analysis on the test dataset with 40 years of age set as the cut-off value. The analytical results confirmed no statistically significant age differences across the study groups, with detailed statistical data presented in Table 4.

**Table 3:** Performance of the artificial intelligence model for classifying voice disorders under different classification conditions.

Class	Sensitivity	Specificity	Acurrency (%)	F1-Score	Average area under the curve values
2	0.922	0.85	89.5	0.921	0.96
3	0.702	0.86	71.6	0.693	0.85

**Table 4:** Subgroup analysis of age in test dataset.

Age group	N	Acurrency (%)	Macro_Precision	Macro_Recall	Macro_F1	Weighted_F1	Macro_AUC_OVR	*P* value
<40	194	72.1	0.70	0.71	0.70	0.71	0.87	N/A^a^
≥40	130	70.7	0.72	0.71	0.67	0.67	0.80	0.057

a N/A, not applicable.

**Figure 4:**
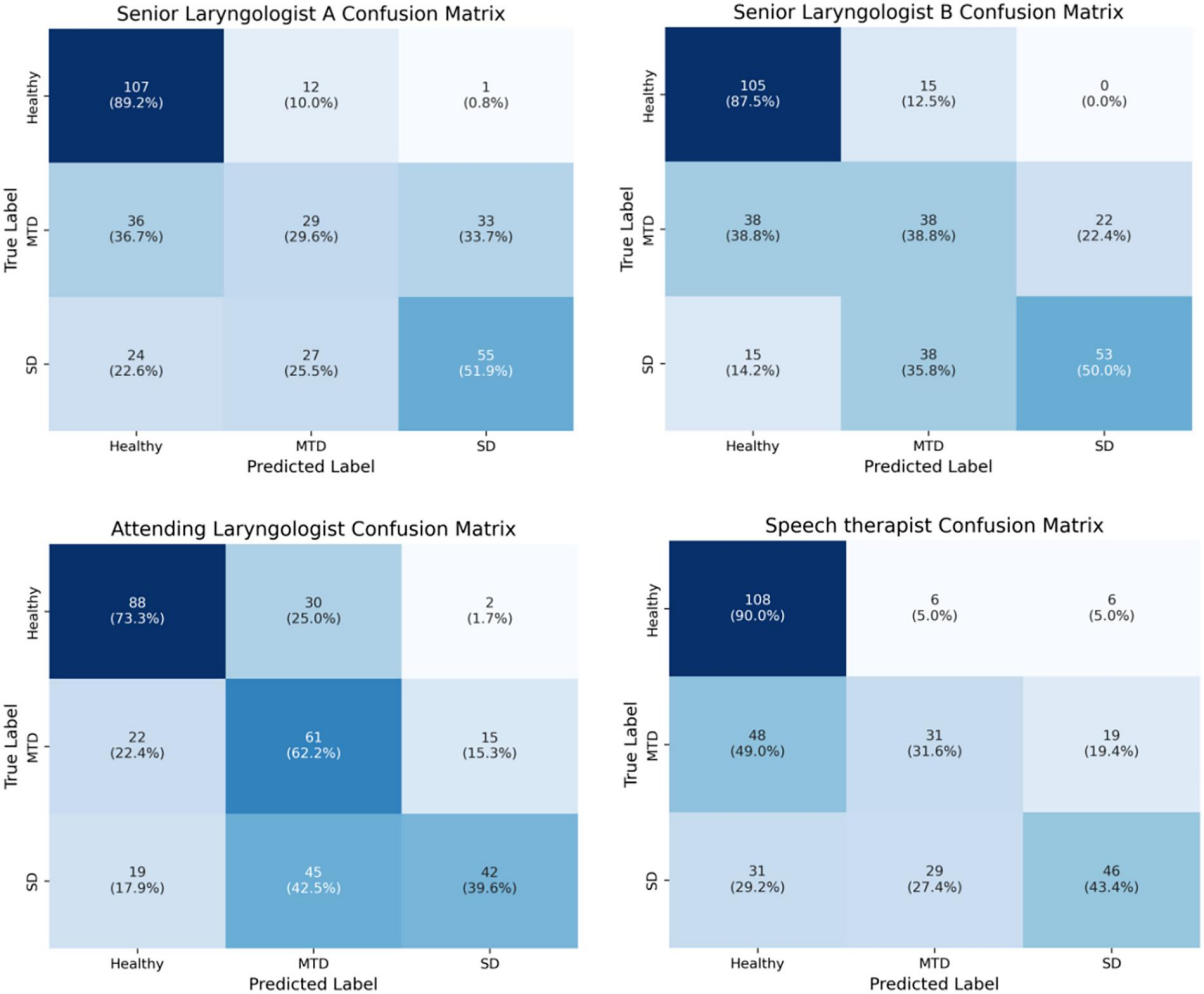
Confusion matrix of 3 classifications in human experts. Healthy = normal voice; MTD, muscle tension dysphonia; SD, spasmodic dysphonia.

Furthermore, we invited four ENT specialists to identify vocal fold pathology by voice using these 3 classifications. The results are shown in Table 5 and Figure 4. The accuracy rates were 59% and 60.5% for the 2 senior laryngologists, 59% for the attending laryngologist and 57.1% for the speech therapist (ST).

**Table 5:** Comparison of the performance for a 3-classification condition by our artificial intelligence model and 4 human experts.

Test participants	Sensitivity (95% CI)	Specificity (95% CI)	Acurrency (95% CI)	*P* value
Model	0.702 (0.654–0.748)	0.858 (0.833–0.882)	0.716 (0.664–0.765)	N/A^a^
Senior Laryngologist A: 20 Y^b^	0.569 (0.520–0.615)	0.792 (0.766–0.818)	0.590 (0.534–0.642)	<0.001
Senior Laryngologist B: 15 Y	0.588 (0.537–0.635)	0.802 (0.774–0.826)	0.605 (0.549–0.657)	<0.001
Attending Laryngologist: 8 Y	0.584 (0.533–0.636)	0.796 (0.770–0.822)	0.590 (0.534–0.642)	<0.001
Speech therapist: 8 Y	0.550 (0.504–0.596)	0.781 (0.756–0.805)	0.571 (0.515–0.623)	<0.001

a N/A, not applicable.

b Y, years of experience in clinical practice.

**Figure 5:**
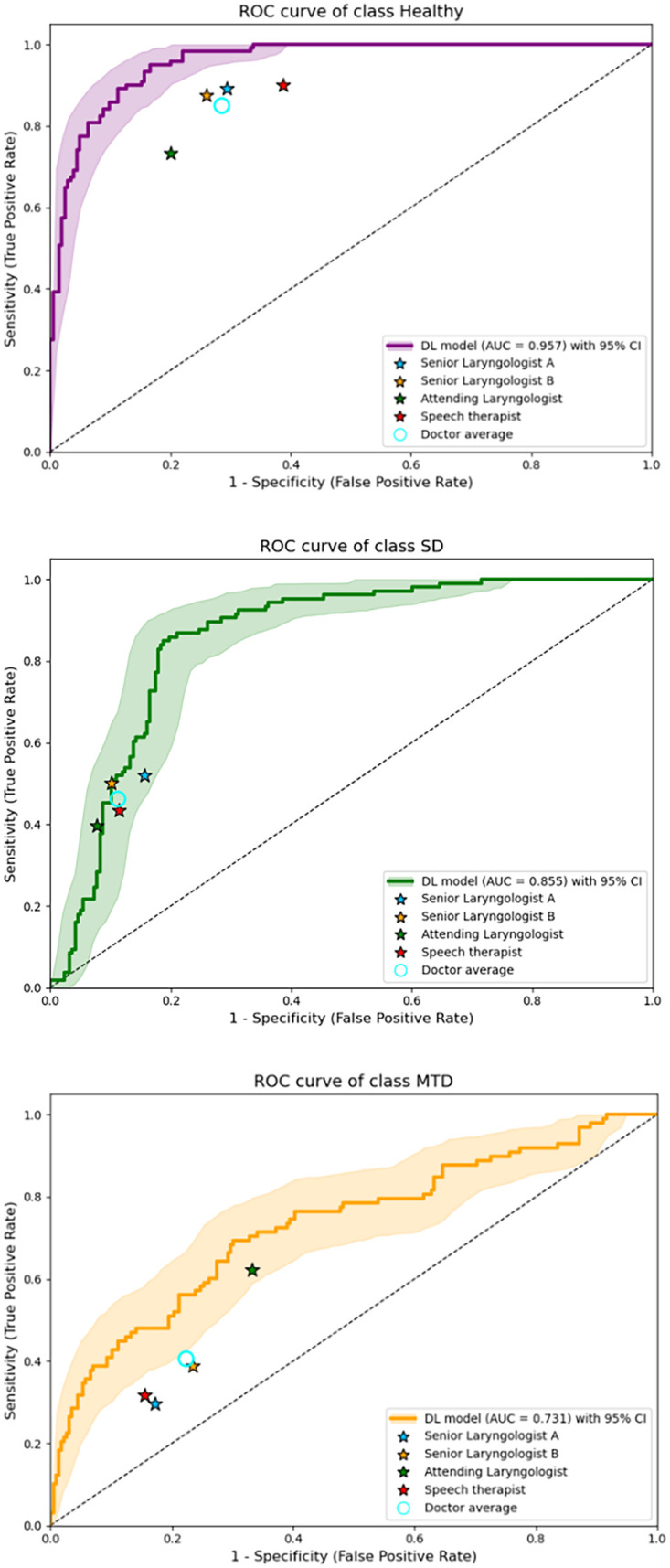
Receiver operating characteristic plots for the benchmark tests. Clinical experts are marked by colored asterisks for individual performance and by an open circle for averaged performance. AUC, area under the curve.

This model also matched or even surpassed the diagnostic capabilities of human experts in this task (Figure 5). In diagnosing SD, it demonstrates slightly better performance than human ears, while in diagnosing MTD and normal voice, its performance is significantly superior to humans. *post hoc* pairwise comparisons revealed that the model excelled over the all human experts (Table 5). Moreover, the proposed model demonstrated perfect consistency, surpassing all human experts who exhibited higher SDs in all outcome metrics and lower scores of intrarater reliability.

**Figure 6:**
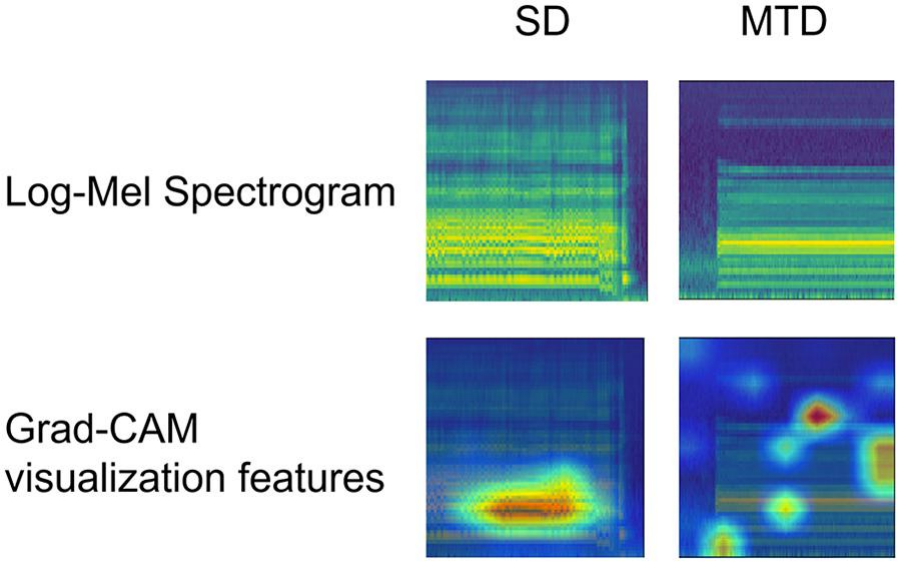
Examples of heat maps. Area marked by hot signals indicate the presence of graphic patterns contributing to a “positive” prediction. MTD, muscle tension dysphonia; SD, spasmodic dysphonia.

Heat Maps from the model consistently highlighted the Log-Mel spectrogram that manifested pathological findings characteristic of the target condition. Figure 6 shows typical heat signal distributions for three types of voice classifications: in normal voice samples, the hot signals are concentrated in high-frequency areas; in SD voice samples, the hot signals are concentrated in low-frequency areas; and in MTD voice samples, the hot signals are scattered.

## 5 Disscussion

### 5.1 Principal findings

Spasmodic dysphonia (SD) is a rare speech disorder characterized by intermittent voice breaks and strained phonation that severely impairs patients' social functioning (42, 43). Unlike most other voice disorders, first-line treatment for SD involves type A botulinum toxin injection into the thyroarytenoid muscle. However, reliable diagnostic tools for SD remain absent, resulting in frequent misdiagnoses and an average 4.4-year delay before appropriate intervention (41). Muscle tension dysphonia (MTD) closely mimics the vocal signatures of adductor spasmodic dysphonia (ADSD), creating frequent clinical confusion. To date, no standalone diagnostic test can reliably differentiate these two conditions under standard clinical criteria (44). Collectively, these diagnostic gaps demonstrate an urgent demand for an affordable, objective auxiliary tool for SD identification.

Our AI model delivers strong performance on binary classification of normal vs. pathological voices, with an AUC of 0.956, consistent with prior relevant artificial intelligence research (30, 31). This robust binary discrimination forms the fundamental screening capacity of our system. For the three-class task (normal voice, SD, MTD), the model yields respective AUCs of 0.731 and 0.855 for MTD and SD differentiation. This moderate multi-class performance aligns with real-world clinical difficulties: auditory-only discrimination between SD and MTD is notoriously unreliable, contributing to widespread misdiagnosis (45, 46). Compared with its excellent binary classification performance, the model's limited three-class accuracy stems from three core barriers: inherent clinical diagnostic ambiguity, limited sample size and class imbalance, and heterogeneous underlying pathophysiology. These confounding factors exert minimal influence on binary discrimination, highlighting the distinct technical challenges of fine-grained multi-subtype voice disorder differentiation.

Specifically, SD and MTD share overlapping clinical, perceptual, and acoustic traits, hindering the model's ability to establish clear feature-space decision boundaries. By contrast, binary classification relies on distinct acoustic markers (e.g., fundamental frequency stability) with minimal overlap, enabling easier learning of robust boundaries. Compounding this, SD's low incidence made data collection difficult: despite 12 years of accumulation, we only obtained 531 SD cases. Binary classification benefits from a larger combined pathological case pool, providing sufficient data for deep learning—whereas limited SD samples restrict three-class performance and prevent segmentation of SD subtypes (ADSD, glottal over-adduction; ABSD, glottal under-adduction) (45, 46). Such subtype analysis is unnecessary in binary tasks, reducing learning burden and improving stability.

Additionally, MTD's acoustic variability (due to patient-specific compensatory mechanisms) further complicates three-class classification. In binary tasks, the model only needs to distinguish “pathological” from normal voices—simpler than differentiating MTD from SD. Collectively, these multi-class-specific factors likely explain the three-class model's lower accuracy compared to our high-performing binary system.

To evaluate the clinical value and decision-aiding capacity of our three-class AI model, we benchmarked its diagnostic accuracy against practicing otolaryngology specialists. This comparative analysis had three primary objectives: (1) quantify the model's performance against human auditory evaluation; (2) examine whether AI could lower variability in subjective perceptual judgment; (3) verify its feasibility as an auxiliary clinical screening tool. On the ternary classification task, the model attained an overall accuracy of 71.6%, while individual clinicians reached 59% (senior laryngologist A), 60.5% (senior laryngologist B), 59% (attending laryngologist) and 57.1% (speech therapist). The AI also displayed comparative strengths in recognizing pathological vocal samples. We additionally observed higher diagnostic accuracy among senior laryngologists relative to less experienced clinicians, indicating accumulated clinical experience improves identification of abnormal voice characteristics. Overall, the model achieved competitive classification performance relative to human raters. It holds promise to assist clinical specialists by reducing subjective evaluation discrepancies and acting as an objective preliminary screening aid, particularly in settings with limited access to senior voice disorder experts.

As shown in Table 6, human raters exhibited poor intra-rater consistency when reassessing identical voice samples across separate time points, a limitation not present in the deep learning model. In terms of operational efficiency, four specialists spent several hours evaluating the 324 test voice recordings, while the AI completed the full dataset inference within seconds, highlighting its practical merit for high-throughput screening workflows. The divergent clinical experience levels among the four participating clinicians further explained the low inter-rater diagnostic consistency observed in human assessments.

**Table 6:** Intraclass correlation coefficient(ICC) results of human experts.

Test participants	Senior laryngologist A 1	Senior laryngologist B 1	Attending laryngologist 1	Speech therapist 1
Senior Laryngologist A 2	0.642	0.683	0.760	0.385
Senior Laryngologist B 2	0.693	0.721	0.685	0.386
Attending Laryngologist 2	0.619	0.709	0.756	0.498
Speech therapist 2	0.703	0.691	0.698	0.384

1:The first test of human experts.

2:The second test of human experts.

We convert raw voice recordings into Log-Mel spectrograms through sequential preprocessing: resampling, pre-emphasis, framing, windowing, Fourier transformation, amplitude squaring, Mel filtering, and logarithmic power computation, generating one spectrogram per audio sample. The Log-Mel scale mimics human auditory perception, closely aligning with subjective pitch sensation and faithfully reflecting acoustic physical properties (47). We adopt Grad-CAM heatmaps to interpret the model's feature-learning patterns (37). Heatmap activation signals reveal distinct acoustic signatures across voice categories and offer objective evidence for differential diagnosis of dysphonia.

Normal voices show concentrated high-frequency activation, clearly distinguishable from pathological signals. Spasmodic dysphonia (SD) exhibits dominant low-frequency heatmap responses, consistent with prior acoustic studies (48). Wide blank bands on SD spectrograms correspond to recurrent vocal fold hyperadduction during phonation, accompanied by abrupt frequency jumps, intermittent voice breaks, and aperiodic noise; such abnormalities are most pronounced in adductor spasmodic dysphonia (ADSD). By contrast, abductor spasmodic dysphonia (ABSD) displays intense activation within breathy segments featuring glottal insufficiency, while mixed SD combines strained and breathy activation alongside scattered high-energy hotspots. Consistent with clinical observations (49), Mandarin third tones (the lowest fundamental frequency among four Mandarin tones) readily induce vocal fold hyperadduction in SD patients — a pattern fully recapitulated in our heatmaps, confirming the clinical validity of Grad-CAM visualization. Muscle tension dysphonia (MTD) presents diffusely distributed high-frequency activation, representing excessive supraglottic tension driven by compensatory muscle overactivity. Its prominent subharmonic and noise-region signals reflect pathological mechanisms fundamentally different from SD.

Unlike previous studies that relied on handcrafted acoustic descriptors such as MFCCs or cepstral coefficients with traditional machine-learning classifiers, the present work leverages CNN-based transfer learning to automatically learn high-level time–frequency features from Log-Mel spectrograms. This approach provides a data-driven representation of subtle dysphonic patterns and extends prior pathology detection research to the inter-pathological differentiation between MTD and SD, which is both clinically significant and technically more challenging.

This study validates an audio-only AI model for screening and differential diagnosis of SD and MTD with promising performance across our study cohorts. In auditory-only diagnostic tasks that pose clinical perceptual difficulties, the model achieves classification accuracy comparable to experienced human clinicians when distinguishing SD from MTD. Furthermore, our interpretable heatmap visualization makes the model's decision-making process traceable and improves its clinical transparency. This AI pipeline can automatically extract key acoustic features and support rapid dysphonia assessment with minimal manual workload for clinicians.

### 5.2 Limitations

This study has several limitations that should be acknowledged. First, although the dataset was collected from two hospitals in different cities, the overall sample size remains relatively small, and the participants are limited to populations in southern China. Such constraints may restrict the broad applicability of the proposed models and weaken their generalizability to wider demographic groups. To resolve this issue in future work, we will expand our study to recruit multi-center cohorts from hospitals across diverse provinces in China, enroll participants speaking different dialects and languages, and conduct independent external validation using fully unseen datasets, so as to further improve the robustness and extrapolation ability of our research outcomes. Second, the current model was trained exclusively on audio samples for analysis. However, a comprehensive diagnosis of voice disorders typically requires the integration of multi-source information, including patients' medical history, clinical symptoms, laryngoscopic findings, auditory perceptual assessments, and acoustic analysis. Relying solely on audio data fails to fully exploit the potential of artificial intelligence (AI) in holistic voice disorder evaluation, which may limit the model's clinical utility in complex diagnostic scenarios. Finally, the dataset used in this study featured a nearly balanced distribution of Muscle Tension Dysphonia (MTD, *n* = 471) and Spasmodic Dysphonia (SD, *n* = 531) cases, which minimized concerns related to class imbalance. Although SpecAugment-based time and frequency masking was used to improve robustness against temporal and spectral perturbations, the present study did not explicitly evaluate model performance under real-world background noise or device variability. Future studies should include noise-corrupted test samples and prospectively collected real-world recordings to further assess deployment robustness.

### 5.3 Future research

Building on the current findings and addressing the identified limitations, future research will focus on three key directions to advance the application of AI in voice disorder diagnosis. First, efforts will be directed toward leveraging emerging technologies to enhance model performance, with effectiveness evaluated through larger-scale controlled trials. This includes exploring advanced deep learning architectures and validating their performance in multi-center settings to ensure consistency across clinical environments. Second, a broader, nationwide dataset will be compiled by integrating patient data from hospitals across China. This expanded dataset will enable systematic assessment and optimization of the models' generalization capabilities, particularly in adapting to diverse patient populations (e.g., varying age groups, comorbidity profiles) and different acoustic collection devices. Third, our follow-up work will focus on developing multimodal AI models that integrate multi-source clinical information—such as medical history, laryngoscopic results, auditory assessments, demographic information, symptom data, and other medical records—generating diagnostic predictions through dedicated fusion layers. This design not only mimics the holistic decision-making strategies of human clinicians but also has the potential to significantly enhance model robustness; additionally, we will expand the scope of human evaluators to include emergency department triage physicians, primary care practitioners, and non-otolaryngology clinicians for more comprehensive validation.

In the next phase of research, multi-center, prospective clinical trials will be conducted to evaluate the practical value of these AI models in real-world clinical settings, with a focus on metrics like diagnostic consistency with expert consensus and improvements in clinical workflow efficiency. Subsequently, the deep learning model will be translated into practical tools: one direction is the development of a real-time voice diagnosis system for outpatient use, enabling clinicians to obtain preliminary screening results during patient consultations; another is the creation of a mobile health (mHealth) application that serves as a preliminary screening tool for pathological voice signals in patients with suspected voice disorders. The ultimate goal of this research program is to establish a reliable AI system that assists clinicians in achieving accurate, efficient, and transparent evaluation and management of voice disorders.

## 6 Conclusions

This study makes notable contributions to the field of acoustic-based voice disorder diagnosis, with its core value reflected in three key aspects. First, it develops and validates an AI framework that relies exclusively on voice signal detection to conduct preliminary acoustic differentiation between SD and MTD—filling the gap in existing research that often focuses on binary normal/pathological classification rather than specific subtype differentiation. It should be noted that since the model is based solely on acoustic information without integrating multimodal clinical data, its differentiation effect is an initial acoustic-based analysis rather than a comprehensive clinical differential diagnosis. Second, the proposed model exhibits promising acoustic differentiation performance. In our internal test set, its predictive capacity is comparable to that of experienced clinical experts, demonstrating the potential of acoustic AI as an auxiliary objective screening tool for voice disorders. Third, the study highlights the model's expandable performance potential: continuous accumulation and incorporation of additional multi-source clinical acoustic data may further improve its accuracy and stability, laying a foundation for iterative optimization and real-world deployment as an acoustic preliminary screening aid.

Beyond its academic contributions, the model delivers tangible practical merits for clinical workflows. Serving as an auxiliary preliminary screening tool for SD and MTD, it targets prominent bottlenecks in current clinical practice: automated voice signal analysis substantially shortens the turnaround time required for preliminary subtype discrimination, which previously depended on labor-intensive manual auditory evaluation and serial clinical examinations. This approach helps mitigate heavy workloads for otolaryngologists and speech-language pathologists amid high clinical volumes, and streamlines the management workflow of voice disorder patients—especially valuable for medical institutions and regions with insufficient specialized voice care resources. For patients, this automated pipeline enables convenient, rapid preliminary acoustic assessment, which may reduce risks of delayed intervention stemming from long waiting periods for specialist evaluation or inconsistent subjective auditory judgment.

Ultimately, this study constitutes an exploratory stepping stone toward embedding AI tools into routine voice disorder evaluation. With follow-up efforts to address the present study's limitations and implement the outlined future research plans, refined AI systems may assist clinicians by supplying objective, time-efficient acoustic references, potentially optimizing standardized voice disorder assessment and benefiting patient care in broader clinical contexts.

### 6.1 Attachment

For the ensemble framework, three CNN-based submodels (VGG16, ResNet50, and DenseNet161) were trained under identical configurations implemented in PyTorch. Each submodel was fine-tuned with a batch size of 4 for 200 epochs using the AdamW optimizer (initial learning rate = 9 × 10^‒7^) in conjunction with a OneCycleLR scheduler (max_lr = 9 × 10^‒7^, pct_start = 0.3, div_factor = 2, anneal_strategy = “linear”) to ensure smooth and stable convergence. The cross-entropy loss function was employed for optimization.

To mitigate overfitting, an early stopping mechanism based on validation accuracy was applied, whereby training was automatically terminated when accuracy failed to improve over a predefined number of epochs. During inference, the softmax probabilities of the three submodels were averaged to generate the final ensemble prediction.

## Data Availability

The raw data supporting the conclusions of this article will be made available by the authors, without undue reservation.
